# Household wellbeing and health risks in Mexican households with and without migrants: a cross-sectional analysis

**DOI:** 10.1186/s40985-018-0096-5

**Published:** 2018-08-01

**Authors:** René Leyva-Flores, Juan Pablo Gutierrez, Cesar Infante, Tonatiuh Gonzalez-Vazquez, Laura Magaña-Valladares

**Affiliations:** National Institute of Public Health, Av. Universidad 655, Santa Maria Ahuacatitlan, 62100 Cuernavaca, Morelos Mexico

**Keywords:** Emigration and immigration, Health profile, Health services accessibility, Mexico

## Abstract

**Background:**

Migration between Mexico and the USA constitutes the world’s largest migration corridor with more than 13 million movements of people in 2016. Furthermore, Mexico has a complex migration profile, being a country of origin, transit, destination, and return. While there has been discussion on the relationship between migration and development of origin communities, evidence on social and health issues faced by origin households is limited. This case study is a first attempt at documenting, through analyzing a national representative health survey of Mexican households (*n* = 9474), the relationship between international migration from Mexico and origin household health characteristics.

**Case presentation:**

Mexican international migration moves largely (90% of migrants) toward the USA. Migration has passed from being mostly circular (from the early to late 1990s) to a permanent pattern of residence in the destination country due to changes in migration policies that have progressively restricted the irregular entrance of immigrants making re-entry more difficult.

The present case study compares the socioeconomic, demographic, and health characteristics of households in Mexico with and without emigrants using data from a national representative health survey. Accordingly, in 2016, 5.8% (*n* = 1,802,980) of all Mexican households reported having a member living abroad.

Households with members living abroad were found to more likely be headed by a female (45.8%), have *Seguro Popular* health insurance, and not to be among the poorest household population. In terms of health profile, a higher frequency of adults with a reported diagnosis of diabetes and/or hypertension (33.9 vs 21.7% for households with vs without emigrants, respectively; *p* = 0.067), and a higher severity of diabetes reflected a higher probability of hospitalization.

**Conclusions:**

Results showed that socioeconomic, demographic, and health conditions differed between households with and without emigrants. These differences were determined as not being attributable to migration and cannot be considered as predisposing factors of migration.

## Background

International migration is a key element of socioeconomic development and health at the global level, particularly for developing countries [1]. The outcomes of international migration may be positive or negative for society, having been documented both in the case of health of transit migrants and destination countries, as well as in returning migrants and their origin households and communities [2, 3].

At a global level, research on international migration has been focused on the relationship between monetary remittances and poverty, suggesting that migration is inversely related to the level and severity of poverty in origin countries [4, 5]. Moreover, Adams and Page [4] suggested that a 10% increment in the percentage of international migrants would lead to a decline of up to 2.1% of the number of people who live with less than US$1 per person per day.

Most remittances are sent to those who are left in charge of the household, typically women; being a female-headed household raises that household’s probability of receiving remittances by 33% [6]. These remittances are used to improve household living conditions and education and for acquiring health insurance [6–12].

Despite the relevance of migration for social, economic, and health statuses, literature on the relationship between migration and health is limited [13–15]. Regarding health, research has been focused on migrants at destination countries, but few studies have focused on understanding how international migration affects or is related to the health of the family who is left behind in the country of origin [2, 3].

From these limited studies, evidence suggests differences in health conditions at the household level compared with households with and without migrants in the same localities. Salgado et al. found that women of migrants’ families in rural communities from three Mexican states reported a higher frequency of chronic and sexually transmitted diseases [16]. Baker [17] found that children of migrants´ families had a higher body mass index and were at risk of being overweight compared with those without migrant relatives; furthermore, migrant families’ obesity levels and patterns were more likely to be similar to those of Mexican Americans in the USA than to those of families with non-migrants in Mexico.

Ullmann et al. [18] analyzed the health status of 2121 men who were the head of households in 14 communities in Mexico and reported that, compared with those with no migratory experience, those with migratory experience had a higher prevalence of obesity (22.8 vs 19.0%, respectively), diabetes (12.0 vs 9.9%, respectively), hypertension (17.3 vs 13.1%, respectively), cardiovascular diseases (6.9 vs 3.6%, respectively), and emotional and/or psychiatric disorders (13.1 vs 5.9%, respectively).

Beslau et al. reported differences in conduct disorder among Mexican emigrants living in the USA and their families in Mexico, suggesting that for some health conditions, living environment is more relevant than genetic influence, in particular for non-aggressive behavior compared with aggressive behavior [19]. Borges et al. found that while no differences existed in terms of alcohol consumption or abuse between the Mexican immigrants in the USA and Mexicans in Mexico, the immigrants were at higher risk for drug use than Mexicans in Mexico [20].

Although these studies provided evidence on the differences in health outcomes at the household level when comparing households with and without members who were abroad, the overall panorama of the relationship between migration and health conditions at the household level remains unclear. In this case study, we discuss the differences of chronic diseases and risks between Mexican households with and without migrants, illustrating how migration affects the health of origin households. Because of chronic conditions, particularly diabetes and hypertension, which are the main drivers of the burden of disease in the country, the analysis focuses on these conditions.

## Methods & Case presentation

A cross-sectional analysis comparing households based on their report of having members abroad was implemented using the data from the National Health and Nutrition Survey 2016 (ENSANUT 2016, according to its Spanish acronym). The ENSANUT 2016 is a national and regional representative probabilistic survey of Mexican households living in non-collective dwellings, with urban and rural areas being represented among four geographical regions of Mexico. For the ENSANUT 2016, the data were collected from a sample of 9474 households, encompassing 29,795 dwellers. A complete description of ENSANUT 2016 methodology is reported elsewhere [21].

The data on household and dwelling characteristics were obtained from a key informant from the household, usually the head of the household or their spouse. Additionally, the data were collected from one household member according to the following age groups: 5–9 years (school-age children), 10–19 years (adolescents), and ≥ 20 years (adults). These individuals were randomly selected from the list of household members who were living in the household at the time of the interview. Specifically designed questionnaires for each age group were created.

As an innovation for health surveys in Mexico, the ENSANUT 2016 included a set of questions regarding whether or not any member of the household immigrated to an international destination. For the analysis in this paper, the data regarding emigrants in the household, household demographics, and socioeconomic status, as well as data on the health conditions of other adults living in the households, were taken from a representative sample of individuals aged ≥ 20 years (*n* = 8824).

Because not all household members were interviewed, the analysis regarding health conditions from household members assumed that the adults were randomly selected as representative conditions in the households.

### Variables used

For all households, members who had migrated abroad and were still considered a part of the household were identified. Questions regarding their main sociodemographic characteristics and destination countries were asked. A dichotomous variable was created using this information to classify households as either with or without international emigrants. Likewise, households with emigrants were categorized according to those with emigrants in the USA or other countries.

To generate a profile of the households’ general characteristics, the following sociodemographic variables were included: female-headed households (with male-headed households as a reference), ethnic condition (comparing those who self-identified as indigenous vs those who did not), social insurance status of the head of the household (using no insurance as a reference compared with having social security or having only *Seguro Popular* health insurance), and the number of schooling years of the head of the household (computed from the level and grade reported as last finalized) and the number of residents and number of children. For socioeconomic indicators, the analysis included vehicle ownership, computer ownership, and number of rooms in the household.

As a socioeconomic indicator, income quintiles were imputed using a methodology proposed for the ENSANUT 2012 and published elsewhere [22], using the National Households Income and Expenditure Survey 2014 (ENIGH 2014, according to its Spanish acronym) as a reference information source. In brief, the procedure first identified common variables in both the ENIGH 2014 and the ENSANUT 2016, which were then applied to the ENIGH 2014 to predict income quintile. Following this, the next stage coefficients from that prediction were used in the ENSANUT 2016 to impute quintiles.

Two variables were used for ethnic status: if the head of the household spoke an indigenous language (with the reference being spoke no indigenous language) and if that household head considered themselves to be indigenous (with the reference being reporting to not be indigenous). Health insurance was analyzed as having any insurance or none and classifying insurance by social security or by *Seguro Popular* affiliation, a government-funded scheme with a pre-defined set of covered interventions. The social security system provides health insurance among other services for those working on the formal market and their families, while the Seguro Popular was created to provide health insurance to the large percentage of the population lacking access to social security.

The data from the adults’ questionnaire were used to identify households with at least one individual with a previous diagnosis of diabetes or hypertension. Furthermore, among households with at least one person with diabetes or hypertension, those who were admitted to emergency services or who were hospitalized within the last year were identified. Households with at least one adult person who had smoked at least 100 cigarettes in their life and with at least one adult person who was currently smoking were also identified. For all of these variables, the reference category was the interviewed adults who did not report these conditions.

### Analysis

A descriptive analysis was carried out considering survey design and weights to compare households with and without international emigrants. In addition, a logistic regression analysis was implemented to identify the correlation between health conditions and having a household member residing abroad. Health conditions served as dependent variables and existence of emigrants as an independent variable, with adjustments made according to socioeconomic quintile and social insurance status. Analyses were performed using Stata 14 (StataCorp, College Station, TX, USA). In these models, the dependent variables were proxy measures for household conditions and derived from the reports of adult individual interviews.

The ENSANUT 2016 was reviewed and approved by the International Review Board of the National Institute of Public Health, Mexico. Informed consent was obtained from participants before the survey was conducted.

## Results

From a total of 31.3 million households in Mexico, 5.8% (representing 1,802,980 households) reported having at least one family member living abroad at the time of the survey. Of these, 91% were in the USA. These households had an average of 1.4 emigrants, of which 1 = male and 0.4 = female, with an average age of 39.5 years.

Table 1 shows the general sociodemographic characteristics of the households, thereby contrasting households with and without emigrants. Up to 45.8% of households were female-headed, with that proportion rising in emigrants’ households to 52.8% (the difference was not statistically significant).

**Table 1:** Sociodemographic characteristics of Mexican households reporting at least one emigrant as a household member in 2016 according to percentage (95% confidence interval)

Variables	All households	Households without an emigrant	Households with one or more emigrants	*p*
Female head of household	45.48	45.02	52.89	0.167
	(42.67–48.28)	(42.26–47.78)	(41.64–64.14)	
Head of household speaks an indigenous language	7.54	7.74	4.17	*0.005*
	(5.60–9.48)	(5.80–9.69)	(1.18–7.16)	
Head of household self-identifies as an indigenous person	29.13	29.06	30.40	0.755
	(26.59–31.68)	(26.47–31.64)	(21.98–38.82)	
Head of household knows how to read and write	88.75	88.78	88.19	0.789
	(87.33–90.16)	(87.34–90.22)	(83.87–92.51)	
Vehicles in the household	0.47	0.47	0.59	0.661
	(0.40–0.55)	(0.39–0.54)	(0.38–0.81)	
Number of residents in the household	3.03	3.04	2.83	*0.032*
	(2.95–3.12)	(2.96–3.13)	(2.64–3.02)	
Computer in the household	28.45	28.66	25.03	0.335
	(25.21–31.69)	(25.43–31.90)	(17.09–32.97)	
Number of rooms in the household	3.53	3.5	3.92	*0.068*
	(3.41–3.64)	(3.39–3.62)	(3.47–4.36)	
Years of schooling of the head of household	7.24	7.25	7.21	0.874
	(7.05–7.44)	(7.06–7.44)	(7.05–7.72)	
Number of children in the household	1.21	1.22	1.04	*0.008*
	(1.15–1.27)	(1.16–1.28)	(0.91–1.17)	
Head of household with health insurance	87.00	87.00	86.97	0.994
	(85.60–88.40)	(85.50–88.50)	(80.25–93.69)	
Head of household without health insurance	13.00	13.00	13.03	0.994
	(11.60–14.40)	(11.50–14.50)	(6.31–19.75)	
Head of household with social security	44.05	44.80	31.74	*0.007*
	(41.23–46.88)	(41.72–47.89)	(23.51–39.98)	
Head of household with *Seguro Popular*	42.95	42.20	55.23	*0.021*
	(39.95–45.95)	(39.11–45.29)	(44.59–65.87)	
Household in the 1st quintile SE	13.43	13.78	7.69	*0.000*
	(11.65–15.20)	(11.95–15.61)	(4.76–10.63)	
Household in the 2nd quintile SE	16.06	16.20	13.83	0.234
	(14.63–17.49)	(14.74–17.66)	(9.97–17.70)	
Household in the 3rd quintile SE	21.70	21.28	28.64	0.307
	(19.15–24.26)	(18.94–23.62)	(14.14–43.13)	
Household in the 4th quintile SE	25.30	25.33	24.70	0.854
	(22.93–27.66)	(22.95–27.72)	(17.86–31.55)	
Household in the 5th quintile SE	23.51	23.41	25.13	0.661
	(20.03–26.99)	(19.91–26.91)	(17.00–33.27)	

Source: Own elaboration with data from ENSANUT, 2016

Regarding ethnic condition, households with emigrants reported a lower percentage of the head of households speaking an indigenous language compared with those without emigrants (4.17 vs 7.74%, respectively; *p* = 0.005). Additionally, households with emigrants compared with those without emigrants were observed to have fewer members (2.83 vs 3.04, respectively; *p* = 0.032) as well as children (1.04 vs 1.22, respectively; *p* = 0.008). Regarding head of household being indigenous and being able to read and write, no differences were found. Nor were any differences observed with respect to goods ownership, such as motor vehicles or computers in the household.

Concerning health insurance, differences were not observed in the percentage of those without any type of insurance (13% in both groups of households, i.e., with and without migrants); however, significant differences were evident with regard to the type of health insurance. In households without emigrants, social security affiliation (44.8%) prevailed. However, in households with emigrants, 55.2% were affiliated with *Seguro Popular*. Finally, regarding socioeconomic level, a lower percentage of households was observed to fall in the first quintile (lower income) of emigrants’ households (7.6 vs 13.7%, *p* < 0.001) compared with those without emigrants.

With regard to health conditions, while the prevalence of diagnosed diabetes and hypertension was higher for households with emigrants than those without emigrants, this difference was not significant (Table 2). Overall, 23.1% of emigrants’ households reported at least one person with diabetes and 29.7% reported at least one person with hypertension.

**Table 2:** Health characteristics of Mexican households reporting at least one emigrant as household member in 2016 according to percentage (95% confidence interval)

Variables	All households	Household without emigrant	Household with emigrants	*p*
Previous diagnosis of diabetes	12.02	11.31	23.11	0.141
	(10.49–13.55)	(10.11–12.51)	(7.48–38.75)	
Previous diagnosis of high blood pressure	19.34	18.67	29.76	0.145
	(17.46–21.22)	(16.79–20.56)	(15.27–44.25)	
Diagnosis of diabetes and/or high blood pressure	22.42	21.72	33.91	*0.067*
	(20.53–24.31)	(19.81–23.62)	(21.27–46.55)	
Medical urgency related to diabetes	13.17	13.47	10.86	0.721
	(9.60–16.74)	(9.83–17.11)	(−2.86–24.58)	
Hospitalization related to diabetes	15.84	10.97	53.05	*0.057*
	(6.80–24.88)	(7.79–14.15)	(9.90–96.21)	
Has smoked 100 cigarettes or more	26.54	27.00	19.27	*0.009*
	(24.57–28.51)	(25.07–28.93)	(13.36–25.17)	
Smokes	14.69	14.92	11.07	0.105
	(13.11–16.27)	(13.33–16.51)	(6.41–15.72)	

Source: Own elaboration based on data from ENSANUT, 2016

Households with emigrants reported a higher percentage of at least one person with diabetes and/or hypertension compared with those without emigrants (33.9 vs 21.7%, respectively; *p* = 0.067). Conversely, among households with people with diabetes, hospitalizations were found to occur to a larger percentage among households with emigrants than those without emigrants. Finally, among households with emigrants, a lower percentage of adults reported having smoked (19.2%) compared with those without emigrants (27.0%).

When analyzing the correlation between health conditions and emigration, by controlling for socioeconomic level and insurance condition, households with emigrants presented with greater odds that a household member had been diagnosed with diabetes and/or hypertension (Table 3). In the case of diabetes, the odds ratio was 2.3 (*p* = 0.038), while that for hypertension was 1.9 (*p* = 0.065) and that for diabetes and/or hypertension combined was 1.9 (*p* = 0.022). Moreover, for those households with people diagnosed with diabetes, the presence of emigrants was related to greater odds of hospitalization: 6.7 (*p* = 0.004). Conversely, regarding tobacco use, households with emigrants reported fewer adults having smoked than those without emigrants, with an odds ratio of 0.66 (*p* = 0.016).

**Table 3:** Adjusted odds ratio^1^ (standard error) for reported occurrence of selected health conditions in Mexican households, Mexico, 2016

Variables	Diabetes	High blood pressure	Chronic diseases	Medical urgency related to diabetes	Hospitalization related to diabetes	Ever smoked	Currently smokes
Household with emigrants	2.38** (0.99)	1.90* (0.65)	1.91** (0.54)	0.94 (0.69)	6.73*** (4.44)	0.66** (0.11)	0.71 (0.16)
Without health insurance (reference)	1.0	1.0	1.0	1.0	1.0	1.0	1.0
With social security (including health insurance)	2.29*** (0.38)	1.95*** (0.32)	2.10*** (0.29)	2.03 (0.92)	2.28 (1.29)	1.17 (0.19)	1.17 (0.22)
With health insurance by *Seguro Popular*	1.89*** (0.39)	1.30 (0.22)	1.52*** (0.23)	1.54 (0.71)	2.58* (1.34)	0.87 (0.11)	1.10 (0.16)
1st SE quintile (Reference)	1.0	1.0	1.0	1.0	1.0	1.0	1.0
2nd SE quintile	1.08 (0.20)	1.03 (0.19)	1.03 (0.16)	0.72 (0.38)	1.01 (0.55)	1.23 (0.18)	1.57** (0.34)
3rd SE quintile	1.39 (0.28)	1.70*** (0.31)	1.44** (0.22)	0.69 (0.29)	2.83** (1.35)	1.13 (0.19)	1.28 (0.31)
4th SE quintile	1.45** (0.22)	1.70*** (0.31)	1.54*** (0.23)	1.14 (0.47)	1.17 (0.58)	1.41** (0.24)	1.57* (0.42)
5th SE quintile	0.80 (0.16)	1.05 (0.20)	0.92 (0.16)	0.42* (0.20)	0.72 (0.44)	1.65*** (0.27)	1.65* (0.44)
Constant	0.06*** (0.01)	0.11*** (0.03)	0.14*** (0.03)	0.11*** (0.06)	0.04*** (0.02)	0.28*** (0.04)	0.11*** (0.02)
Observations	8609	8609	9451	986	986	8799	8799

Source: Own elaboration based on data from ENSANUT, 2016

Standard errors in parenthesis *** *p* < 0.01, ** *p* < 0.05, * *p* < 0.1

^1^Multivariate logistic regression, adjusting for the other factors shown in the table

## Discussion

At a global level, international migration is a phenomenon that shapes the contemporary world despite an important strengthening of anti-immigration policies [23]. Migration is a complex phenomenon both in terms of its causes and its consequences for social, economic, and health issues. Therefore, migration represents one of the most relevant challenges for the world and requires the study and design of public policies to be oriented toward the protection and guarantee of human rights of migrants in origin, transit, and destination societies [24].

Mexico is a society with an important migratory experience in terms of origin, transit, destination, and return country of migrants [25], with a strong bond between migrants’ abroad and their families back home. This case study focused on the analysis of these families and how their health status was affected by or related to migration.

We therefore focused on health and sociodemographic conditions of households in Mexico with and without emigrants. Results showed elements that contribute to characterizing the socioeconomic and health profile of households with emigrants differed from those without emigrants. Within emigrants’ households, a female head predominated, and they were not among the poorest households. Furthermore, such households were less likely to be headed by an indigenous language speaker. Additionally, *Seguro Popular* was the predominant health insurance type, suggesting that a larger proportion of emigrants’ households than non-emigrant households had precarious jobs and employment without social security.

Interestingly, households that reported having a member living abroad were determined to also have higher odds of having members in Mexico with a diagnosis of diabetes and/or hypertension. The same households were found to have much higher odds of reporting diabetes-related hospitalizations. These findings combined may reflect more severe conditions of the disease.

Because analysis was cross-sectional, determining differences regarding socioeconomic and health conditions of households with emigrants and those without emigrants prior to migration or after migration was not feasible. Possibly, the higher presence of health conditions might be related to reasons for emigrating, such as ensuring access to resources that provide health care. As has been previously reported, Mexican health services face important challenges that limit their accessibility [26]. In particular, for individuals covered by *Seguro Popular*, a relatively limited coverage of health interventions is offered.

Alternatively, health conditions occurring post-migration may be related to transfer of consumption patterns from those individuals that migrated. This was proposed by Villa et al. [27] who documented the existence of transnational health practices, having both positive and negative impacts on the health of origin households.

Taking into account the evidence presented, establishing a position that leads to a public policy decision that mitigates differences among the analyzed social groups is difficult. This difficulty is particularly pronounced given the limitations of the data and considering that the ENSANUT 2016 is not designed to identify aspects related to migration. The identification of migration characteristics was restricted to the reports of those households surveyed, which did not include specific information about monetary remittances, nor sociodemographic and health characteristics of the emigrants. Therefore, the basic characteristics of the emigrants remain unknown, whether they do or do not send remittances, nor if people in the household have migratory experience. It is worthwhile to note that this was a transversal study that explored, for the first time, the relationship between migration and health in a representative sample at a national level. Therefore, we cannot assume casualty relationships in terms of health attributable to migration nor of health as a predisposing factor of migration when observing higher levels of health damage in emigrant households in origin communities.

## Conclusions

In Mexico, quantitative research on migration and health has been dependent on one of the two approaches. The first is by using different databases to answer a given question. The second approach is by informing decision makers about the complicated endeavor of defining public policies that mitigate inequities in health and access to health care services for mobile populations. Concentrations of studies have focused on the migratory phenomenon, highlighting its positive effects mainly in the case of monetary remittances [13]. Therefore, other implications and the heterogeneous or negative effect of migration in Mexico remain unexplored. In this regard, incorporating variables that explore health issues in population-based surveys or those that address migration is important.

Findings from this case study enable the establishment of critical discussion regarding the relationship of international migration and the socioeconomic development of communities and countries of origin, including how these factors affect health. As Delgado [28] argued, demystifying the positive impacts of migration is important: migration implies diverse economic costs, such as depopulation, diminishment of productive activities, and dependence on remittances for families and locations of origin that are not compensated by the remittances. Furthermore, migrants are exposed to risks that can lead to harm after they leave their country of origin [29–33].

Portes [34] proposed a less pessimistic view, where migration is economically beneficial for most migrants and their families, but noted that no evidence exists for remittances per se being sufficient to promote economic development. Furthermore, migration may have a negative effect on government action. Additionally, poverty eradication cannot depend on currency flows through remittances because most migration flows do not originate from households in extreme poverty [35]. This is important because certain socioeconomic conditions are required to support the migration process and eventually the flow of economic and social remittances.

Mexico is a country with a large population that migrates toward the USA. This movement has resulted in both strong social networks that support migrants and exchange of knowledge and goods as part of an international relationship [36].

The findings from this case suggest that policies aimed at improving the wellbeing (including health) of migrants and their families require public investment to promote social and economic development. In particular, there exists an urgent need to design studies that are able to identify the health consequences of migration on origin households as well as to identify how health conditions (exacerbating demand for resources) may become an incentive for migration.
